# Catestatin Regulates Epithelial Cell Dynamics to Improve Intestinal Inflammation

**DOI:** 10.3390/vaccines6040067

**Published:** 2018-09-20

**Authors:** Nour Eissa, Hayam Hussein, Ruth Mesgna, Sandra Bonin, Geoffrey N. Hendy, Marie-Hélène Metz-Boutigue, Charles N. Bernstein, Jean-Eric Ghia

**Affiliations:** 1 Department of Immunology, Max Rady College of Manitoba, University of Manitoba, Winnipeg, MB R3E 0T5, Canada; Nour.Eissa@umanitoba.ca (N.E.); Hayam.Hussein.57@gmail.com (H.H.); ruth25@live.com (R.M.); Boninsandra09@gmail.com (S.B.); 2 Internal Medicine section of Gastroenterology, Max Rady College of Manitoba, University of Manitoba, Winnipeg, MB R3A 1R9, Canada; Charles.Bernstein@umanitoba.ca; 3 IBD Clinical and Research Centre, University of Manitoba, Winnipeg, MB R3A 1R9, Canada; 4 Children’s Hospital Research Institute of Manitoba, University of Manitoba, Winnipeg, MB R3E 3P4, Canada; 5 Department of Parasitology and Animal Diseases, Veterinary Research Division, National Research Centre, Giza 12622, Egypt; 6 Metabolic Disorders and Complications, McGill University Health Centre-Research Institute, Departments of Medicine, Physiology, and Human Genetics, McGill University, Montréal, QC H3A 0G4, Canada; geoffrey.hendy@mcgill.ca; 7 INSERM U.1121, Faculté de Chirurgie Dentaire, Place de l’Hôpital, 67000 Strasbourg, France; marie-helene.metz@inserm.fr

**Keywords:** chromogranin-A, colitis, host-defense peptides, inflammatory bowel diseases, innate immunity, intestinal permeability, mucosal drug action

## Abstract

Ulcerative colitis (UC) is characterized by aberrant regulation of tight junctions (TJ), signal transducer and activator of transcription 3 (STAT3), and interleukin (IL)-8/18, which lead to intestinal barrier defects. Catestatin (CST), an enterochromaffin-derived peptide, regulates immune communication and STAT-3 in the inflamed intestine. Here, we investigated the effects of CST during the development of inflammation using human biopsies from patients with active UC, human colonic epithelial cells (Caco2), and an experimental model of UC (dextran sulfate sodium [DSS]-colitis). In UC patients, the protein and mRNA level of CST was significantly decreased. Colonic expression of *CST* showed a strong positive linear relationship with TJ proteins and *STAT3*, and a strong negative correlation with *IL-8* and *IL-18*. Intra-rectal administration of CST reduced the severity of experimental colitis, IL-18 colonic levels, maintained TJ proteins and enhanced the phosphorylation of STAT3. CST administration increased proliferation, viability, migration, TJ proteins, and p-STAT3 levels, and reduced IL-8 & IL-18 in LPS- & DSS-induced Caco2 cell epithelial injury, and the presence of STAT-3 inhibitor abolished the beneficial effect of CST. In inflammatory conditions, we conclude that CST could regulate intestinal mucosal dynamic via a potential STAT3-dependent pathway that needs to be further defined. Targeting CST in intestinal epithelial cells (IECs) should be a promising therapeutic approach such as when intestinal epithelial cell homeostasis is compromised in UC patients.

## 1. Introduction

The intestinal epithelium incorporates a wide range of dynamic biological processes, including intestinal barrier function, which plays a crucial role in the intestinal homeostasis and inflammatory response [1]. Cell-to-cell junctions, specifically tight junctions (TJ), are essential proteins involved in regulating the epithelial barrier function. Alteration of TJ contributes to the pathophysiology of inflammatory bowel disease (IBD) in both Crohn’s disease (CD) and ulcerative colitis (UC) [2]. Dysregulation of the epithelial barrier integrity is a consequence of an aberrant and continuing inflammatory response due to the release of pro-inflammatory cytokines challenging mucosal homeostasis [3]. TJ proteins are intracellular junctional complexes, and their architecture involves transmembrane proteins and junctional adhesion molecules such as Claudin1 (CLDN1), Occludin (OCLN), and Zona occludens1 (ZO1) proteins, which regulate the paracellular permeability [4]. The loss of TJ proteins aggravates the intestinal inflammation by increasing the influx of luminal antigens in the lamina propria, which amplifies the inflammatory cascades and mediators within the intestinal mucosa [5,6].

Inflammatory mediators in turn regulate the epithelial cell and barrier function through an increase of interleukin (IL)-8 and IL-18 released by the intestinal epithelium. These cytokines mediate wound healing and ultimately barrier function [7]. These signals induce the activation of transcription signaling factors, such as signal transducer and activator of transcription 3 (STAT-3), which has a protective function and promotes mucosal healing [8,9]. STAT-3 plays a major role in epithelial reconstitution as STAT3-deficient mice are highly susceptible to dextran sodium sulfate (DSS) and have impaired wound healing [10]. 

Intestinal epithelial and neuroendocrine cells throughout the GI tract express a large number of peptides that have complementary activities and play fundamental roles in regulating the host-microbe interactions and mucosal immune responses that underlie the pathogenesis of IBD [11,12,13]. In the gastrointestinal tract, enterochromaffin cells secrete the neuroendocrine pro-hormone CHGA [11], which is elevated in patients with IBD and plays a crucial role in the development of IBD [11,14]. Processing of CHGA gives rise to several peptides that play a significant role in intestinal mucosal immunity [6,11,15,16]. We have previously shown that the CHGA-derived peptide, catestatin (CST; (hCHGA_352–372_), protects against the development of acute intestinal inflammation in two murine models of experimental colitis through modulation of pro-inflammatory macrophages function via a STAT3-dependent mechanism [17]. Recently, we have also shown that CST attenuates the severity of chronic colitis relapse by decreasing the activity of M1 but not M2 macrophages [18]. 

In the current study, using human colonic biopsies, a model of dextran sulfate sodium (DSS)-induced colitis, and a human colonic epithelial cell line, we investigated the relationship between CST and colonic TJ dynamics in the context of inflammation. 

## 2. Material and Methods

### 2.1. Human Subjects

This study was approved by the University of Manitoba Health Research Ethics Board (HS14878 [E]). Endoscopic colonic biopsies were collected from ten patients with active UC and ten persons with no evidence of IBD (healthy individuals). Patients and healthy individuals were recruited from the University of Manitoba IBD Clinical and Research Centre. Mean age for control and active UC groups were 50.30 ± 12.86 (*n* = 10) and 39.90 ± 12.98 (*n* = 10), *p* < 0.05, respectively. Informed consent was obtained from patients and control subjects. 

### 2.2. Mice

Six to eight-week-old male C57BL/6 mice (20–25 g body weight) purchased from Charles River (Sherbrooke, QC, Canada) were maintained in the University of Manitoba specific pathogen-free barrier animal care facility. Experiments were conducted under the Canadian guidelines for animal research and approved by the University of Manitoba Animal Ethics Committee (Protocol # 15-010).

### 2.3. Peptides

Human CST (CST) (hCHGA352-372: SSMKLSFRARAYGFRGPGPQL) was purchased from Biopeptide Co., Inc., San Diego, CA, USA. The selected experimental dose of 1.5 mg/kg/day was based upon previously published data on the use of peptides for intra-rectal (*i.r.*) injection [16,17,18,19]. Control groups received *i.r.* injection of 1× phosphate buffer saline (PBS). In the context of acute colitis, our previous studies [17] had demonstrated the specificity of CST, and the use of a scrambled peptide was omitted in the present study.

### 2.4. Acute DSS-Induced Colitis

Intra-rectal injection of CST (1.5 mg/kg/day) or 1× PBS started one day before colitis induction was given for five days. DSS (molecular weight [MW], 40 kDa: MP Biomedicals, Soho, OH, USA) was added to the drinking water at a final concentration of 5% (*w*/*v*) for five days (37) to 6–8-week-old mice. DSS was freshly dissolved every two days. Controls were time-matched with mice receiving regular drinking water only. Mean DSS consumption was noted per cage each day. 

### 2.5. Assessment of DSS-Induced Colitis 

To assess the severity and onset of colitis, weight loss, stool consistency, and bleeding were recorded from day zero up to day five during DSS treatment [6]. Previously we showed that CST reduced the macroscopic score and histological damage in acute DSS-induced colitis [17]. In the current study, we report the macroscopic and histological scores to confirm the reduction of inflammatory cascades in the colon. Mice were sacrificed at day five, and macroscopic scores were evaluated. Histopathological scores, formalin (Sigma, Mississauga, ON, Canada)-fixed colon segments from the splenic flexure were paraffin (Sigma)-embedded, and 3 mm sections were stained using hematoxylin eosin (H&E) (Sigma). Histologic damage was assessed according to a published scoring system that considers structural damage, goblet cell depletion, edema/ulceration, and degree of inflammatory cells infiltrate [6].

### 2.6. Enzyme-Linked Immunosorbent Assay (ELISA)

Epithelial associated cytokines (IL-8, IL-18) and IL-6 (R&D Systems, Inc., Minneapolis, MN, USA), CST (CUSABIO, Cedarlane, Burlington, ON, Canada) and phosphorylated-STAT3 (STAT3 [pY705] ELISA, LifeScience, Burlington, ON, Canada) quantification were performed on clarified full-thickness colon homogenates from mice and or supernatants or cell lysates from Caco-2 cell culture using specific mouse and human commercial ELISA kits.

### 2.7. Caco-2 Epithelial Cell Culture and Treatment with CST

Human intestinal epithelial cell line, Caco-2 (ATCC, Manassas, VA, USA), was cultured and handled as described previously [6] in Eagle’s Minimum Essential Medium (EMEM) (glutamine, high glucose) supplemented with 100 U/mL penicillin, 100 μg/mL streptomycin, and 20% heat-inactivated FBS. Cell culture medium was changed every three days until the cells fully differentiated (80–90% confluent). For each experimental setup, three separate experiments were performed, with at least six wells per condition. Caco-2 cells were seeded at 3 × 10^5^ cells/well in culture plates and treated with 2 ml of medium containing CST (100 ng/mL) or 1× PBS for 24 h. Then, cells were challenged with LPS (1 μg/mL) or 5% DSS for an additional 24 h in the presence or absence of STAT3 V blocker (10^−5^ M, STAT Three inhibitory compound [STATTIC]; Sigma). Gene and/or protein expression of TJ proteins (*CLDN*, *OCLN*, *ZO*), IL-8, IL-18, p-STAT3 were quantified, and migration, proliferation, viability, and oxidative stress survivability of Caco-2 cell line were evaluated as previously described [6].

### 2.8. Quantitative Real-Time Reverse-Transcription Polymerase Chain Reaction 

Total RNA was extracted from human biopsies, mice colonic tissue, and Caco-2 cells using TRIzol™ Plus RNA Purification Kit (Life Technologies, Grand Island, NY, USA) and reverse transcribed using SuperScript VILO cDNA Synthesis Master Mix (Invitrogen, Grand Island, NY, USA). A quantitative real-time reverse-transcription polymerase chain reaction (qRT-PCR) was used to quantify gene expression in a Roche light cycler 96 Real-Time System using Power SYBR green master mix (Life Technologies, Burlington, ON, USA). Differences in the threshold cycle (ΔCt) number between the target genes and mouse eukaryotic elongation factor 2 (*Eef2*) and human TATA-box binding protein (*TBP*) (optimal reference genes) [19,20,21] were used to normalize expression. Human and mouse primer sequences for the target gene markers are provided in Table 1 and Table 2. 

### 2.9. Western Blotting

The experiments were performed as previously described [6,14,22]. Briefly, colonic tissue was lysed in RIPA buffer (50 mmol/L Tris pH 8, 0.1% SDS, 0.5% deoxycholate, 1% NP-40, 150 mmol/L NaCl, 1 tablet complete mini protease inhibitor/10 mL, (Roche Diagnostics GmbH, Mannheim, Germany). Then, equal amounts of total lysates were electrophoresed and transferred to Trans W membrane. Membranes were incubated overnight at 4 °C with anti-total STAT3 and anti-p-STAT3 antibodies (Cell Signal, Beverly, MA, USA) at dilution rate 1:1000. GAPDH served as a loading control (1:10,000 dilution). HRP-conjugated anti-rabbit (Cell Signaling) secondary antibodies (1:5000 dilution) were used for chemiluminescent detection. Mini-PROTEAN TGX precast gels (Bio-Rad, Mississauga, ON, Canada) were used for Western blot analysis. Antibodies were purchased from R&D Systems, Mississauga, ON, Canada.

### 2.10. Statistical Analysis

Data are expressed as the mean ± standard error of the mean (SEM). Statistical analyses were performed using an unpaired Mann-Whitney U test, and one- and two-way ANOVA, followed by a post-hoc test when appropriate. Spearman’s correlation test was also used. The statistical two-tailed significance level was *p* < 0.05. Statistics were computed using GraphPad Prism software (version 6; GraphPad Software, Inc., La Jolla, CA, USA).

## 3. Results

### 3.1. CST Peptide Protein Levels Correlate Positively with mRNA Gene Expression of TJ Proteins and STAT3, and Negatively with IL-8 and IL-18 in Patients with Active UC

**Figure 1:**
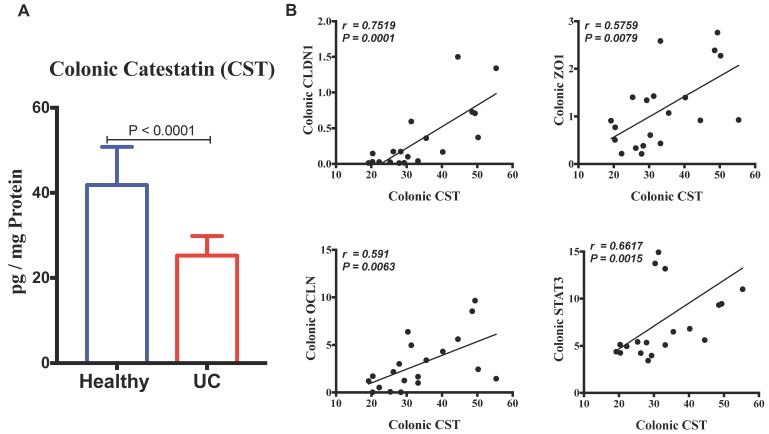
Colonic protein level of Catestatin (CST) has a strong positive linear relationship with mRNA gene expression of tight junction (TJ) proteins and STAT3 in samples pooled from patients with active ulcerative colitis (UC) and healthy individuals. (**A**) The expression levels of CST in patients with UC and healthy individuals; (**B**) The correlation of *CST* with mRNA levels of (Claudin (*CLDN1*), zonula occludens-1 (*ZO1*), and occludin (*OCLN*) and *STAT3*). Spearman’s correlation tests, two tailed significance level adjusted at *p* < 0.05, and Student-*t* test.

**Figure 2:**
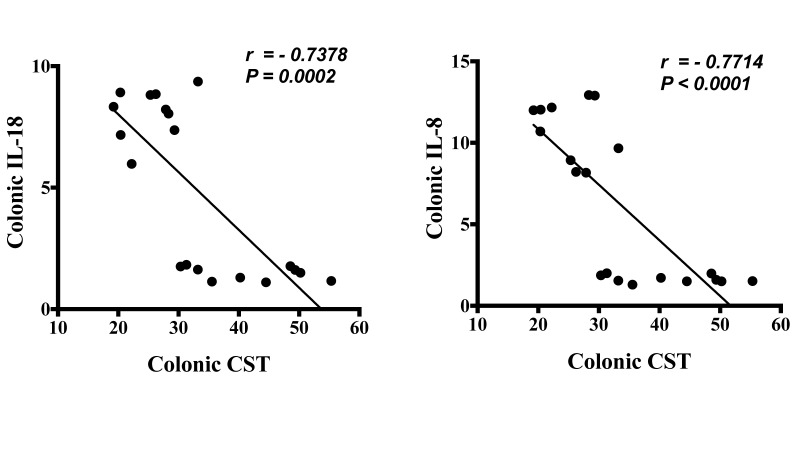
Protein level of Catestatin (CST) has a strong negative linear relationship with mRNA gene expression of epithelial associated cytokines in pooled samples from patients with active ulcerative colitis (UC) and healthy patients. The correlation of CST protein levels with mRNA levels of interleukin (*IL*)*-8* and *IL-18*. Spearman’s correlation, two tails significance level adjusted at 0.05.

First, we investigated the association between protein level of CST peptide and mRNA levels of human pathophysiological markers implicated in active UC. Protein level of CST peptide is reduced in the colonic biopsy specimens of patients with active UC compared to those of healthy individuals (Figure 1A). The protein level of CST was strongly positively correlated with mRNA levels of TJ proteins, *CLDN1*, *OCLN*, and *ZO1*, and *STAT3* (Figure 1B). Conversely, the mRNA levels of epithelial-associated cytokines demonstrated a significant negative correlation with protein level CST (Figure 2). 

### 3.2. CST Attenuates the Onset and Severity of Acute DSS-Induced Colitis

**Figure 3:**
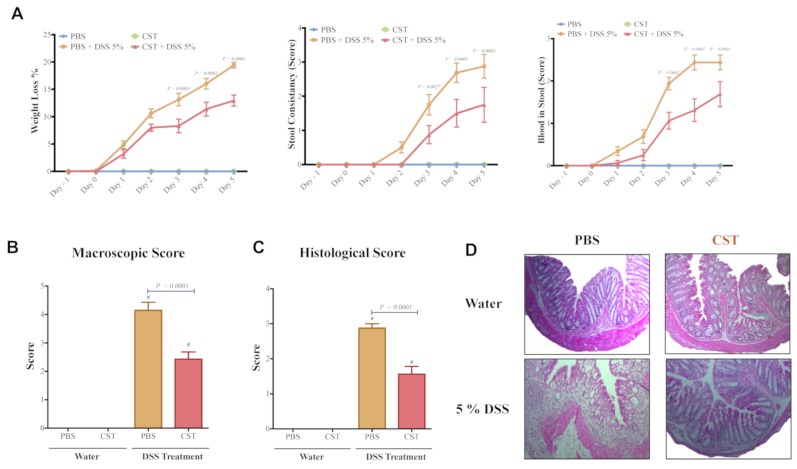
Catestatin (CST) peptide treatment ameliorates DSS-induced colitis. C57BL6 mice were given 5% DSS solution in their drinking water to induce colitis. Control mice received water without dextran sulfate sodium (DSS). Preventive CST (1.5 mg/kg/day) peptides treatment or phosphate buffer saline (1× PBS) started 1 day before colitis induction and lasted for 5 days. (**A**) Weight loss, stool consistency, and blood in stool; (**B**) macroscopic score; (**C**) histologic scores; and (**D**) hematoxylin and eosin (H&E)-stained colonic sections (100× magnifications) on day five after administration of DSS and control groups. Two-way repeated measures or One-way ANOVA followed by multiple comparison tests were used to analyze the data. Each value represents the mean ± SEM, *n* = 7–10 mice/group. ^#^ *p* < 0.0001 compared to control water groups.

First, we confirmed that preventive i.r. administration of CST to DSS-treated mice significantly reduced the onset and severity of colitis (*p* ≤ 0.0001), represented by a decrease in weight loss, stool consistency, and colonic bleeding (Figure 3A). In colitic mice, CST treatment significantly decreased the macroscopic scores compared with colitic PBS-treated mice (Figure 3B). Moreover, CST treatment significantly reduced the histologic score, manifested by a reduction in the loss of tissue architecture and cellular infiltrate (Figure 3C,D).

### 3.3. CST Increases the Activation of STAT3, Maintains Colonic Gene Expression of TJ Proteins and Decreases Colonic IL-18 Release in Acute DSS-Induced Colitis

**Figure 4:**
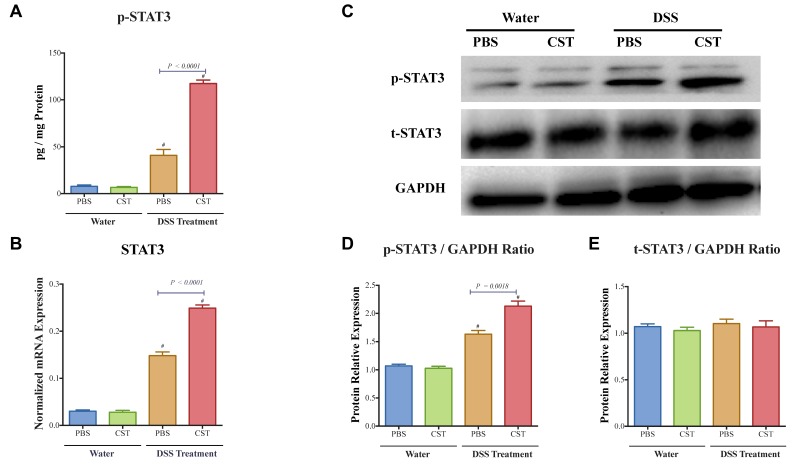
Catestatin (CST) increases the activation of p-STAT3 in dextran sulfate sodium (DSS)-induced colitis. Treatment with CST (1.5 mg/kg/day) or 1× phosphate buffer saline (PBS) started one day prior to DSS colitis induction. (**A**) Colonic phosphorylated levels of p-STAT3 that were quantified using ELISA; and (**B**) colonic mRNA levels expression of *STAT3* were quantified using q-RT-PCR. (**C**–**E**) Colonic total and phosphorylated levels of p-STAT3 that were quantified using western blot. One-way ANOVA followed by multiple comparison tests. Each value represents the mean ± SEM, *n* = 7–10 mice/group. ^#^ Refers to significance compared to control groups.

**Figure 5:**
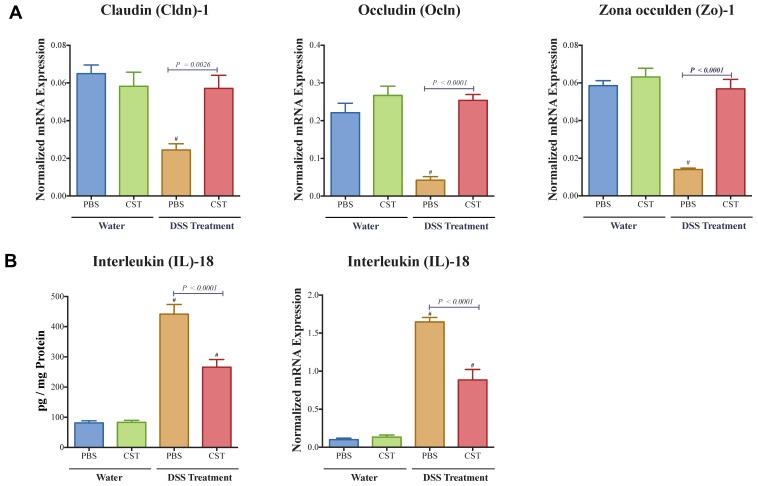
Catestatin (CST) maintains colonic mRNA levels of tight junction (TJ) proteins and reduces interleukin (IL)-18 release in dextran sulfate sodium (DSS)-induced colitis. Treatment with CST (1.5 mg/kg/day) or 1× phosphate buffer saline (PBS) started one day prior to DSS colitis induction. (**A**) Colonic mRNA levels expression of TJ proteins (Claudin (*Cldn1*), zonula occludens-1 (*Zo1*), and occludin (*Ocln*); (**B**) Colonic protein and mRNA levels expression of *Il-18*. One-way ANOVA followed by multiple comparison tests. Each value represents the mean ± SEM, *n* = 7–10 mice/group. ^#^ Compared to control water groups.

As previously shown by ELISA, CST increased the colonic level of p-STAT3 [17]. Here, in addition of the level of p-STAT3, gene expression of *Stat3* was also quantified. CST treatment led to an increase of colonic p-STAT3 level (Figure 4A) and *Stat3* mRNA levels (Figure 4B) in DSS-induced colitis when compared with the DSS control group. Moreover, in colitic condition CST treatment increased the levels of p-STAT3 but did not demonstrate a significant effect on the total protein levels of STAT3 (Figure 4C–E). In parallel, colitic mice exhibited a significant reduction in colonic mRNA levels of TJ proteins (*Cldn1*, *Ocln*, *Zo1*) when compared with non-colitic mice (Figure 5A). Administration of CST abolished this deleterious effect and maintained the colonic expression of TJ proteins (Figure 5A). Moreover, colitic mice also demonstrated an up-regulation of the colonic protein and mRNA levels of *Il-18* (Figure 5B), and CST treatment significantly decreased these levels (Figure 5B). 

### 3.4. CST Enhances the Phosphorylated STAT3 (p-STAT3) in LPS- and DSS-Stimulated Caco-2 Epithelial Cells, But Not in Non-Inflammatory Conditions

**Figure 6:**
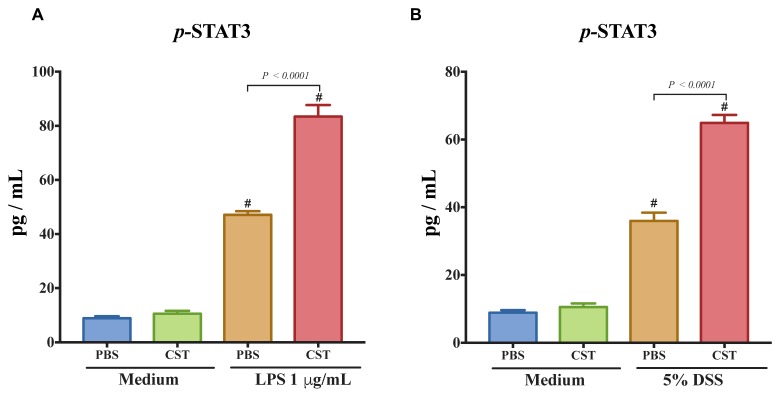
Catestatin (CST) induces the phosphorylation of signal transducer and activator of transcription 3 (STAT-3) in lipopolysaccharide (LPS)- and dextran sulfate sodium (DSS)-stimulated colonic epithelial cell lines. Caco-2 cells were treated with 1× phosphate buffer saline (PBS) or CST (100 ng/mL) in medium for 24 h then challenged with LPS (1 μg/mL) or 5% dextran sulfate sodium (DSS) for additional 24 h. (**A**,**B**) p-STAT3 levels in LPS- and DSS-stimulated epithelial cells. One-way ANOVA was used to analyze the data followed by multiple comparison tests. Data represent mean ± SEM (*n* = 6–9). ^#^ Compared to control medium groups. Each experiment was repeated at least three times.

To confirm the importance of p-STAT3 on epithelial cells, we quantified the level of p-STAT3 in LPS- and DSS-stimulated Caco2 cells. LPS and DSS treatment increased the level of p-STAT3. Treatment with CST significantly increased the phosphorylation levels of STAT3 in both LPS- and DSS-stimulated epithelial cells when compared with inflammatory PBS groups (Figure 6A,B). In the absence of LPS or DSS, CST did not have any effect on the level of p-STAT3 (Figure 6A,B). 

### 3.5. CST Maintains Gene Expression of TJ Proteins and Reduces IL-8 and IL-18 Release in LPS- and DSS-Stimulated Caco-2 Epithelial Cells through an Undetermined Mechanism Involving STAT3, But Not in Non-Inflammatory Conditions

**Figure 7:**
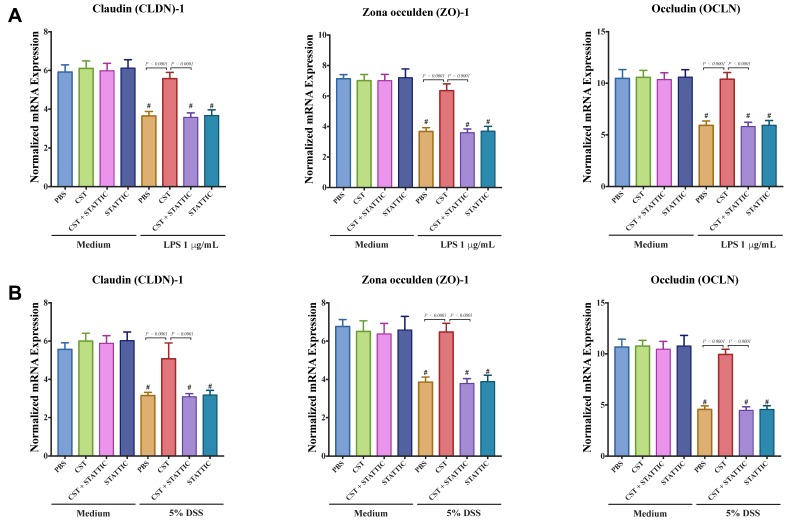
Catestatin (CST) maintains gene expression of TJ proteins in LPS- and DSS-stimulated Caco-2 epithelial cell lines through potentially the STAT3 pathway. Caco-2 cells were treated with 1× phosphate buffer saline (PBS) or CST (100 ng/mL) in medium for 24 h then challenged with LPS (1 μg/mL) or 5% DSS for an additional 24 h in the presence or absence of STAT3 inhibitor (10^−5^ M, STATTIC; Sigma, Mississauga, ON, Canada). (**A**,**B**) mRNA levels expression of TJ proteins (Claudin (*CLDN1*), zonula occludens-1 (*ZO1*), and occludin (*OCLN*) in LPS- and DSS-stimulated epithelial cells. One-way ANOVA was used to analyze the data followed by multiple comparison tests. Data represent mean ± SEM (*n* = 6–9). ^#^ Compared to control groups. Each experiment was repeated at least three times.

**Figure 8:**
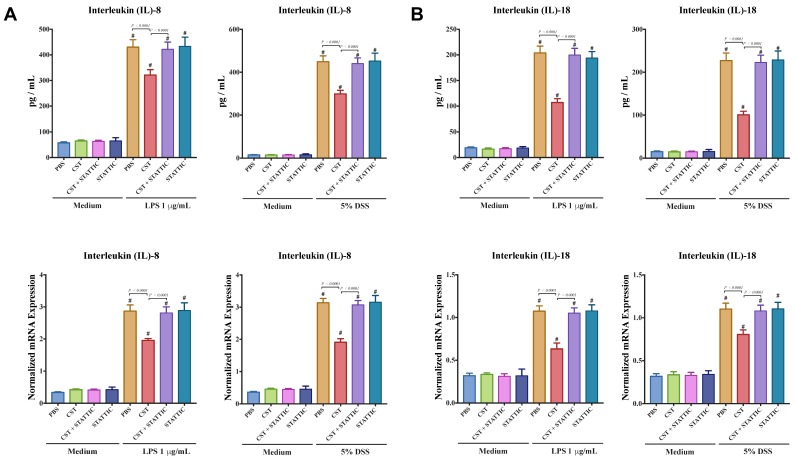
Catestatin (CST) reduces IL-8 and IL-18 release in LPS- and DSS-stimulated Caco-2 epithelial cell line through potentially the STAT3 pathway. Caco-2 cells were treated with 1× phosphate buffer saline (PBS) or CST (100 ng/mL) in medium for 24 h then challenged with LPS (1 μg/mL) or 5% DSS for an additional 24 h in the presence or absence of STAT3 inhibitor (10^−5^ M, STATTIC; Sigma, Mississauga, ON, Canada). (**A**,**B**) Protein and mRNA levels expression of IL-8 and IL-18 in LPS- and DSS-stimulated epithelial cells. One-way ANOVA was used to analyze the data followed by multiple comparison tests. Data represent mean ± SEM (*n* = 6–9). ^#^ Compared to control groups. Each experiment was repeated at least three times.

Next, we explored the effect of CST on the gene expression of TJ proteins, IL-8, and IL-18 in LPS or DSS-stimulated Caco2 cells. LPS and DSS treatment significantly decreased the mRNA levels of TJ proteins (*CLDN1*, *ZO1*, *OCLN*) (Figure 7A,B) and increased IL-8 and IL-18 (Figure 8A,B). CST treatment abolished the deleterious effect induced by LPS and DSS treatments (Figure 7 and Figure 8). Furthermore, inhibition of the STAT3 pathway through the use of STATTIC abolished the beneficial effect of CST in LPS- and DSS-stimulated epithelial cells (Figure 7 and Figure 8). STATTIC treatment alone did not show any effect on LPS or DSS treatments alone. In the absence of LPS or DSS, CST or STATTIC treatments did not demonstrate any effect on IL-8 and IL-18 release or mRNA levels of TJ proteins (Figure 7 and Figure 8).

### 3.6. CST Enhances the Functional Capacities of Colonic Epithelial Cells Including Epithelial Migration, Proliferation, Viability, & Oxidative Stress Resistance.

**Figure 9:**
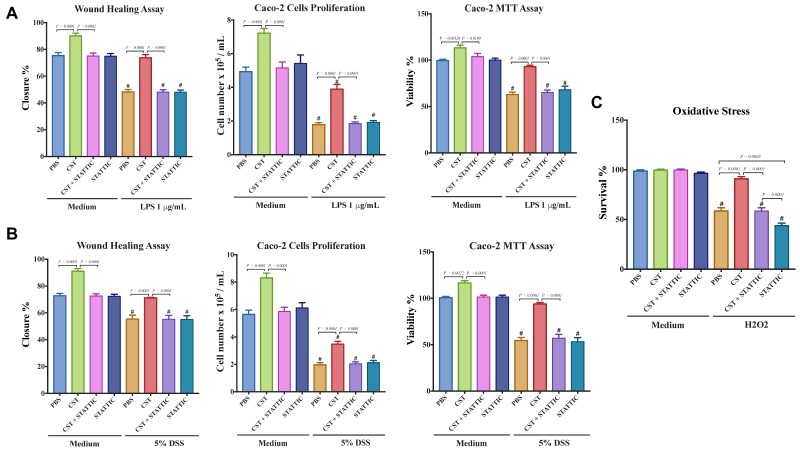
Catestatin (CST) enhances migration, proliferation, and viability of a colonic cell line in inflammatory conditions. Caco-2 cells were pretreated with 1× phosphate buffer saline (PBS) or CST (100 ng/mL) for 24 h, then challenged with lipopolysaccharide (LPS) (1 μg/mL) or 5% DSS) for an additional 24 h in the presence or absence of a STAT3 inhibitor (10^−5^ M, STATTIC; Sigma, Mississauga, ON, Canada). (**A**,**B**) Epithelial cell migration assessed by the wound healing assay, intestinal epithelial cell proliferation, and epithelial cell viability assessed by the 3-(4,5-dimethyl thiazolyl-2yl)-2,5-diphenyl tetrazolium (MTT) assay; (**C**) Epithelial cells oxidative stress assay show survival data from cultures treated with normal medium (control) or 200 mmol/L H_2_O_2_ in the presence or absence of a STAT3 inhibitor (10^−5^ M, STATTIC; Sigma, Mississauga, ON, Canada). One-way ANOVA was used to analyze the data followed by multiple comparison tests. Data represent mean ± SEM (*n* = 6). ^#^ Compared to control groups. Each experiment was repeated at least three times.

Finally, we investigated the effect of CST on the functional capacities of LPS- and DSS-stimulated Caco2 cells. LPS- and DSS-stimulated cells showed a significant decrease in the migration, proliferation, and viability (Figure 9A,B), and CST treatment abolished this effect (Figure 9A,B). Surprisingly, in non-inflammatory conditions, CST-treated epithelial cells exhibited a significant increase in migration, viability, and proliferation (Figure 9A,B). Additionally, we quantified oxidative stress. H_2_O_2_ treatment significantly decreased Caco2 survivability when compared with control conditions, and CST treatment significantly improved it (Figure 9C), however, no effect of CST was visible in control conditions. For all parameters studied, at the exceptions of the oxidative stress, STATTIC treatment alone did not show any effect on LPS or DSS treatments. However, in inflammatory conditions the beneficial effect of CST treatment was abolished in the presence of STATTIC (Figure 8A–C).

### 3.7. CST Decreases IL-6 Release in Acute DSS-Induced Colitis and DSS-and LPS-Stimulated Caco-2 Epithelial Cells, But Not in Control Conditions

**Figure 10:**
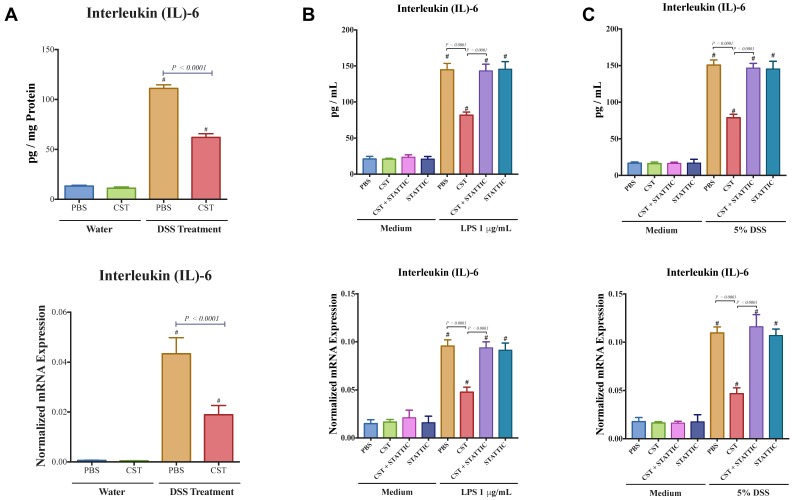
CST decreases IL-6 release in acute DSS-induced colitis and DSS- and LPS-stimulated Caco-2 epithelial cells. (**A**) Colonic protein and mRNA expression of interleukin (IL)-6 of C57BL6 mice were given 5% DSS solution in their drinking water to induce colitis. Control mice received water without dextran sulfate sodium (DSS). Preventive CST (1.5 mg/kg/day) peptides treatment or phosphate buffer saline (1× PBS) started 1-day before colitis induction and lasted for 5-days. Caco-2 cells were treated with 1× phosphate buffer saline (PBS) or CST (100 ng/mL) in medium for 24 h then challenged with LPS (1 μg/mL) or 5% DSS for an additional 24 h in the presence or absence of STAT3 inhibitor (10^−5^ M, STATTIC; Sigma, Mississauga, ON, Canada); (**B**,**C**) The expression of IL-6 in DSS- & LPS-stimulated epithelial cells. One-way ANOVA was used to analyze the data followed by multiple comparison tests. Data represent mean ± SEM (*n* = 6–8). ^#^ Compared to control groups.

Colitic mice also demonstrated an up-regulation of the colonic protein and mRNA levels of *I*l6 (Figure 9A) and CST treatment significantly decreased it (Figure 10A). Next, we explored the effect of CST on the expression of IL-6 in LPS or DSS-stimulated Caco2 cells. LPS and DSS treatment significantly increased IL-6 (Figure 10B,C) and CST treatment abolished this deleterious effect (Figure 10B,C). Moreover, inhibition of the STAT3 pathway abolished the beneficial effect of CST in LPS- and DSS-stimulated epithelial cells (Figure 10). In the control conditions (in absence of LPS or DSS), CST or STATTIC treatments did not show any effect on IL-6 (Figure 10).

## 4. Discussion

The recent demonstration of macrophage-mediated STAT3 regulation of acute colonic inflammation by CST [17] led to the current study. Here, we identified, for the first time, a potential novel mechanism by which CST ameliorates the severity and onset of colitis through regulation of TJ dynamics and intestinal epithelial cells (IEC) homeostasis via a STAT3-dependent pathway. Using an experimental animal model, administration of CST protected against acute DSS-induced colitis by reducing epithelial-associated cytokines and maintaining IECs homeostasis. Furthermore, CST mediated colonic gene expression of TJ proteins, decreased epithelial-associated cytokines, IL-8 and IL-18, release in LPS- and DSS-stimulated Caco2 epithelial cells. The protective features of CST were also exhibited through an increase in migration, viability, proliferation, and oxidative stress resistance of the epithelial cells. Consistent with these experimental results, in patients with active UC, CST was positively correlated with mRNA levels of TJ and STAT3 proteins and a negatively correlated with IL-8 and IL-18. These findings highlight the importance of gut peptides as host-defense peptides, and their role in regulating the intestinal inflammation and wound healing processes [23,24]. Furthermore, this study suggests that the CHGA-derived peptide, CST, contributes to the pathophysiology of IBD by controlling cell/cell junction dynamics, intestinal epithelial cell functions, and restoration of the epithelial barrier.

TJs are highly dynamic components of intestinal barrier function, creating a boundary between the apical and basolateral membranes and acting as a crucial barrier to the diffusion of solutes and luminal content through the intercellular space [25]. The assembly, disassembly, and degree of sealing of TJ vary according to the nature of stimuli and pathological conditions [26]. In this study, we showed that CST correlates positively with TJ proteins and STAT3 and negatively with epithelial-associated cytokines (IL-8 and IL-18) in patients with active UC. TJ proteins and STAT3 are essential components in mucosal healing and tissue remodeling [1,27], which are altered in UC patients [1,27]. But the role of STAT3 is ambiguous, depending of its spatio-temporal expression and activation it can demonstrate different effects [27]. Human epithelial associated cytokines (IL-8 and IL-18) are upregulated and correlated with the clinical severity of IBD [28,29]. Similarly, UC has been characterized by an alteration of the colonic expression of CHGA and some of its derived peptides. This alteration is associated with a dysregulation of TJ proteins [6,11,14,18] suggesting a critical role of these peptides during the development of colitis. Our findings are consistent with the anti-inflammatory properties of CST seen in other inflammatory disorders such as atopic dermatitis [30] and obesity-associated inflammation [31], and the expression of CST is upregulated in the sera of patients with IBD [17]. Surprisingly, in colitic conditions and compared to the serum level [17], our study finds a significant decrease of CST in the colonic mucosa of patients with active UC, suggesting that the level seen in the serum doesn’t reflect the mucosal level. Further studies are in need to define the exact relation between the two spatial differences. 

Building on our previous studies, where we reported a potential protective feature of CST during the progression of acute and chronic colitis [5,16,17,18], our study demonstrates that CST protects against DSS-induced colitis by reducing the expression of IL-18 and maintaining TJ protein expression in the mouse colonic mucosa. IL-18 is a pro-inflammatory cytokine, which amplifies the inflammatory process [32]. In DSS-induced colitis the epithelial barrier defenses are defective, and treatments used to reduce and or to block IL-18, through neutralizing monoclonal antibody or binding protein, induce a protective mechanism associated with a decrease in pro-inflammatory mediators [33,34]. Our data are consistent with the concept that the overall integrity of the intestinal mucosal barrier and TJ proteins is abolished by the induction of the gut inflammation response and our findings are consistent with previously published data demonstrating that reduction of colonic TJ proteins is correlated with the severity of DSS-induced colitis [35]. Maintenance of colonic TJ protein expression is a therapeutic target in preclinical models of IBD. Our study provides a further potential mechanism by which, in inflammatory conditions, CST can protect against intestinal inflammation via the regulation of TJ proteins, as none of these effects were visible in non-inflammatory conditions. 

Activation of STAT3 is associated with enhancement of protective mechanisms in the intestinal mucosa [27]. Previously, we demonstrated that CST was associated with an activation of STAT3 and that CST protected against colitis [17]. In the current study, and although the basal inflammatory event induced an increase of mRNA STAT3 expression and p-STAT3, but not t-STAT3, we demonstrate that CST increased, furthermore, STAT3 and its phosphorylated form in colitic colonic mucosa, and subsequently induces a decrease of associated epithelial cytokines and maintained the expression of TJ proteins. In LPS- and DSS-stimulated human colonic epithelial cells, we found the same effect. In both experimental setups, we demonstrated that the protective effects of CST were abolished by blocking STAT3. Surprisingly, no direct effect of the blockade of STAT3 was seen in Caco2 inflammatory conditions in the absence of CST treatment. Our study showed that CST increased the mRNA expression of STAT3 but not the total protein of STAT3, this could be attributed to other levels of regulation between transcript and protein product [36]. These data align with studies that demonstrated that IEC-specific STAT3 knockout mice were more susceptible to DSS-induced colitis [37,38], suggesting that CST, by regulating the levels of STAT3 signalling in IECs, may limit the extent of mucosal inflammation and allow better mucosal healing and repair responses following an inflammatory insult. 

Aberrations in cell junctions and aberrant epithelial cell movement and wound healing are critically implicated in the pathophysiology of IBD [1,6,9,26]. In the present study, we demonstrated an essential role of CST in the regulation of epithelial proliferation, viability, cell movement, and wound healing in cell culture in response to injury stimuli such as LPS and DSS. In inflammatory conditions, our pharmacological study demonstrated that the use of STAT3 blocker suppressed the beneficial effect of the CST treatment. Surprisingly, in non-inflammatory conditions CST was able to increase all the markers, and STAT3 treatment suppressed that effect, although in control conditions CST did not increase the level of STAT3 or p-STAT3. Our findings are consistent with the observations that activation of STAT3 resulted in an increase of Caco-2 epithelial cell proliferation [39] and an improvement in mucosal wound healing [10]. Overall, enhancement of the functional capacities of IECs is associated with protection against colitis [6]. Again, here at the exception of the effect on oxidative stress, no direct effect of the blockade of STAT3 was seen in Caco2 inflammatory conditions in the absence of CST treatment. As the precise correlative mechanism of CST-mediated protection of inflammation is not clearly demonstrated, it should be further explored. To fully understand the mechanism of action of CST in intestinal pathogenesis, it is essential to examine the changes in the transcriptome or kinome of CST-treated epithelium. Further studies are required to decipher the signaling cascades involved in CST action.

In this preliminary correlative descriptive study, limitations exist especially regarding the direct or the indirect effect of CST on STAT3. Receptors for CST appear not to exist, rather, sequence similarity of CHGA-derived peptides with cell penetrating peptides [40,41,42] appears to allow CST to enter cells. For that reason, CST can regulate through different mechanisms our readouts (i.e., TJ regulation, inflammation and proliferation) and STAT3 can be one of the proteins implicated in the mechanisms. Moreover, it is also possible that the dose or the time of the STAT3 blocker treatment was not optimal, therefore, dose- and time-response studies should be performed. This is particularly visible in the context of IL-8/18 where, if these cytokines were negatively regulated by STAT3, therefore, the level of these cytokines would have increased furthermore. The same applies for the effect seen on TJ, where inhibition of STAT3 at either control inflammatory conditions or stimulatory inflammatory conditions did not reduce the expression of TJ markers. In this study, we only assessed the mRNA level of TJ proteins based on the notion that “DNA makes RNA makes proteins”, suggesting a direct relationship between mRNA and protein levels [43]. Further studies are required to investigate the precise effects of CST on protein expression and localization of TJ proteins. Finally, in this study for the correlation analysis we assembled all the results together. Given that some analyses can portray, in certain cases, a much lower score in the controls, a linear study should have assessed for correlation within each group. Unfortunately, the number of samples in each group was not sufficient, and absolute values for the two groups were pooled. We cannot rule out the possibility that other potential mechanisms can be harnessed by CST, for example, effects on NF-κB, ERK, PI3K, endoplasmic reticulum stress, and angiogenesis, this can be accounted for the effect seen on IL-8/18.

## 5. Conclusions 

In conclusion, our findings provide novel insights into the protective features of CST against intestinal inflammation through the regulation of TJ dynamics and intestinal cell movement. Thus, laying the groundwork to provide a further understanding of intracellular mechanisms and the intricate signaling network orchestrating the crosstalk between CHGA-derived peptides and intestinal epithelium and STAT3 in the physiology and pathology of the human gut; and the role of CHGA-peptides in host defense. 

## Figures and Tables

**Table 1:** Human primers sequences.

Gene Name	Forward	Reverse
***OCLDN***	ACAAGCGGTTTTATCCAGAGTC	GTCATCCACAGGCGAAGTTAAT
***TBP***	CCCGAAACGCCGAATATAATCC	AATCAGTGCCGTGGTTCGTG
***CLDN1***	AGGTGCTATCTGTTCAGTGATG	TGGCTGACTTTCCTTGTGTAG
***ZO1***	CCAGCCTGCTAAACCTACTAAA	ATCTCTTGCTGCCAAACTATCT
***IL8***	ACTGAGAGTGATTGAGAGTGGAC	AACCCTCTGCACCCAGTTTTC
***IL18***	GCGTCACTACACTCAGCTAAT	GCGTCACTACACTCAGCTAAT
***STAT3***	ACCAGCAGTATAGCCGCTTC	GCCACAATCCGGGCAATCT
***IL6***	CCTGAACCTTCCAAAGATGGC	TTCACCAGGCAAGTCTCCTCA

**Table 2:** Mouse primers sequences.

Gene	Forward	Reverse
***Il18***	GACTCTTGCGTCAACTTCAAGG	CAGGCTGTCTTTTGTCAACGA
***Eef2***	TGTCAGTCATCGCCCATGTG	CATCCTTGCGAGTGTCAGTGA
***Ocldn***	TTGAAAGTCCACCTCCTTACAGA	CCGGATAAAAAGAGTACGCTGG
***Cldn1***	GGGGACAACATCGTGACCG	AGGAGTCGAAGACTTTGCACT
***Zo1***	GCCGCTAAGAGCACAGCAA	TCCCCACTCTGAAAATGAGGA
***Il6***	TAGTCCTTCCTACCCCAATTTCC	TTGGTCCTTAGCCACTCCTTC
